# A tetracycline-inducible Split TurboID system for specific biotinylation and identification of nuclear proteins from HEK293T cells

**DOI:** 10.55730/1300-0152.2734

**Published:** 2025-01-06

**Authors:** Mehmet SARIHAN, Fatma Zehra ÖZEN, Murat KASAP, Gürler AKPINAR

**Affiliations:** Proteomics Laboratory, Department of Medical Biology, Faculty of Medicine, Kocaeli University, Kocaeli, Turkiye

**Keywords:** Split-TurboID, nuclear proteome, organelle proteomics, protein biotinylation, nuclear protein identification, tet-regulated protein expression

## Abstract

**Background/aim:**

To overcome the limitations of conventional organelle isolation methods including low purity, low yield, sample degradation, scalability and the need for multiple centrifugation steps, an improved nuclear protein enrichment approach was developed using the modified Split TurboID biotin ligase enzyme.

**Materials and methods:**

A construct was created in which the N-terminal domain of TurboID, fused to the FK506-binding protein (FKBP) was targeted to the nucleus. This construct was incorporated into a tetracycline-inducible gene expression vector. Similarly, the C-terminal domain of TurboID was fused to the rapamycin-binding domain of mTOR (FRB) and directed to the nucleus. This construct was introduced into a constitutive expression vector. A HEK-293T-TetR+ cell line, stably expressing both fusion proteins, was created. Activation of the N-terminal domain was achieved by tetracycline induction while an active Split-TurboID was formed within the nucleus only after the introduction of rapamycin into the culture medium which facilitated the formation of the FKBP-Rapamycin-FRB complex.

**Results:**

The cells expressed N- and C-termini of Split-TurboID and produced an active biotin ligase enzyme in the nucleus, as demonstrated by Western blot and immunofluorescence microscopy analyses. The active enzyme biotinylated both residential nuclear proteins and the proteins that transiently interact with the nucleus. Enrichment and identification of the biotinylated proteins showed that 1518 proteins were identified, of which 78.4% were localized to or colocalized with the nucleus. Comparison with unenriched samples confirmed higher confidence in identification of resident nuclear proteins. Cross-referencing with the Human Protein Atlas highlighted the limitations of current databases, 820 proteins match known nuclear proteins and 698 have not been previously annotated.

**Conclusion:**

Split-TurboID-based approach effectively minimized background noise arising from nonspecific labeling or imperfect localization and provided an appreciable level of specificity resulting identification of both residential and transiently interacting nuclear proteins.

## Graphical Abstract

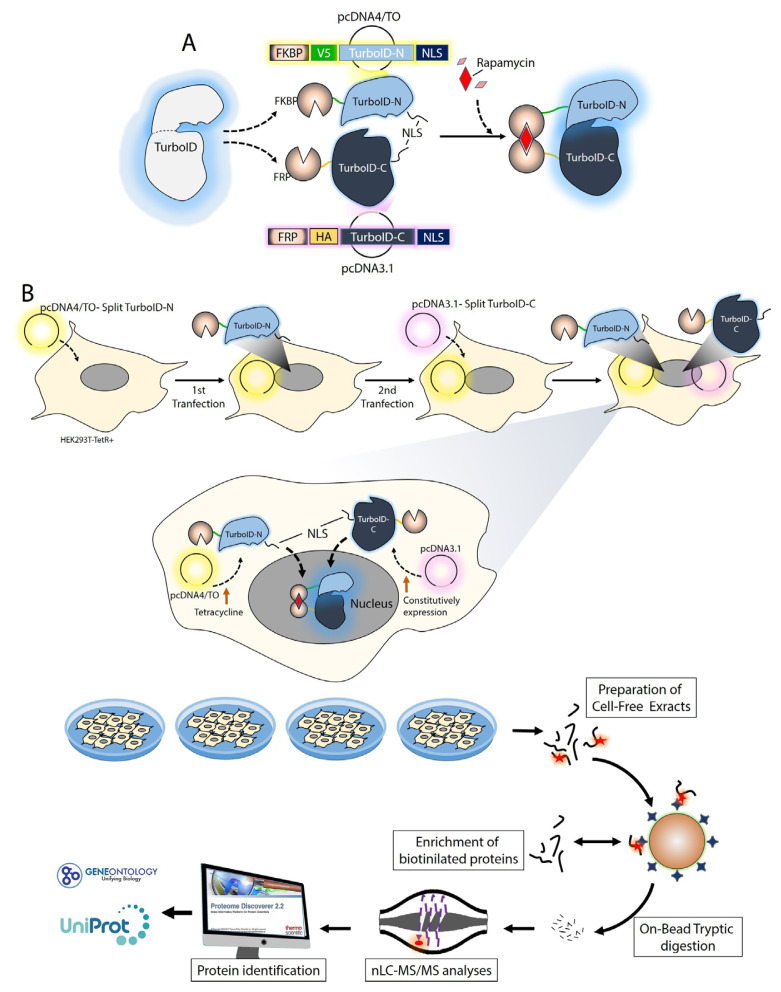

## 1. Introduction

The spatial distribution of proteins at the subcellular level is crucial for understanding protein functions and their interactions with other proteins and cellular components (Qattan et al., 2012). In this context, the Human Protein Atlas (HPA) has been providing valuable information for researchers studying protein localization and expression in human tissues and cells (Lindskog 2016; Thul and Lindskog, 2018). Currently, the expression and localization of more than 65% of human protein-coding genes were determined by immunofluorescence and confocal microscopy in three different cell lines (Thul and Lindskog, 2018). The proteins are localized to 35 different organelles and fine subcellular structures. These findings contributed to a deeper understanding of how proteins form organelles, cells, tissues, and organs (Singh, 2021). However, the methodology used in the HPA project is dependent on the availability and reliability of the antibodies. Although a rigorous antibody validation process is used for selection, the data is not validated by alternative experimental approaches, such as enrichment of subcellular organelles to provide additional experimental evidence. This is in part because organelle enrichment methods have suffered from serious limitations due to the fact that subcellular compartments share similar physical characteristics and can partially cofractionate in conventional gradients (Mueller et al., 2017).

In this study, we interested in enriching nuclear proteins and overcoming the limitations of conventional organelle isolation methods, such as low purity, low yield, sample degradation, the need for multiple centrifugation steps, scalability and the need for specialized instrumentation. For this purpose, we used an approach in which nuclear proteins were biotinylated by Split TurboID, enriched with streptavidin beads and identified with a nHPLC LC-MS/MS system. The N-terminal domain of Split TurboID was fused to FK506-binding protein (FKBP) and targeted to the nucleus using a nuclear localization signal (NLS). The fused genes were cloned into pCDNA 4/TO to achieve tetracycline-inducible gene expression. Similarly, the C-terminal domain of Split TurboID was fused to Rapamycin-binding domain of mTOR (FRB) and targeted to the nucleus using the same NLS. This second set of fused genes was cloned into a constitutive expression vector, pCDNA 3.1. Both vectors were transfected into HEK-293T-TetR+ cells and a stable cell line expressing both proteins was created. Expression of the N-terminal domain was induced by tetracycline. An active Split TurboID was formed in the nucleus only after the addition of rapamycin, which helped formation of active TurboID via the help of FKBP-Rapamycin-FRB complex. In this way, Split TurboID biotinylated the proteins that were localized in the nucleus or colocalized to the nucleus. The biotinylated proteins were enriched with streptavidin-conjugated magnetic beads and identified using a nHPLC LC-MS/MS system. Quantitatively, a total of 1518 proteins were identified in enriched samples. A database search revealed that 1190 (78.4%) of these proteins exclusively localized or colocalized to the nucleus. As shown here, the Split-TurboID-dependent biotinylation approach can be very effective to map proteomes of subcellular organelles.

## 2. Methods

### 2.1. Preparation of recombinant plasmids

FKBP-V5-Split TurboID-N-Terminal-NLS nucleotide sequence was synthesized by Eurofins Genomics (Ebersberg, Germany) and sent to our laboratory as a pEX clone. The sequence was subcloned into pCDNA4/TO using HindIII and BamHI restriction endonucleases. Similarly, the FRB-HA-Split TurboID-C-Terminal-NLS nucleotide sequence was synthesized by Eurofins Genomics and sent to our laboratory as a pEX clone. The sequence was subcloned into pcDNA3.1 using the restriction endonucleases BamHI and EcoRI. The recombinant vectors were then electro-transformed into Escherichia coli XL1 Blue strain and selected with ampicillin. Insert sizes were assessed by agarose gel electrophoresis after restriction digestion.

### 2.2. Establishment of a stable cell line expressing Split TurboID -N and -C terminals

A stable HEK293T-TetR+ cell line was created that expressed FKBP-V5-Split TurboID-N-Terminal-NLS protein under tetracycline control and FRB-HA-Split TurboID-C-Terminal-NLS protein constitutively. To create this cell line, HEK293T-TetR+ cells (ThermoScientific, USA) were first transfected with the pcDNA4/TO-FKBP-V5-Split TurboID-N-Terminal-NLS plasmid clone using the Neon transfection system and selected with zeocin (50 ug/mL). A monoclonal colony was obtained and shown to express the protein of interest by western blotting using an antibody against V5-tag. The pCDNA 3.1-FRB-HA-Split TurboID-C-Terminal-NLS plasmid clone was transfected into this cell line and the cells were selected with geneticin (700 ug/mL). A monoclonal colony was obtained and shown to express the protein of interest by western blotting using an antibody against HA-tag. These cells were grown under selective conditions and used throughout the study.

### 2.3. Cell culture

Hek293T-TetR+ cells expressing Split TurboID N and C termini were grown in DMEM supplemented with 10% Tetracycline-reduced FBS (fetal bovine serum), 100 μg/mL penicillin-streptamycin, 2 mM L-Glutamine, 10 μg/mL Blasticidin, 50 μg/mL Zeocin and 700 μg/mL geneticin at 37 °C and 5% CO_2_ atmosphere. The cells were passaged, frozen and thawed following the standard cell culture protocols. Frozen cells were routinely maintained in liquid nitrogen.

### 2.4. Formation of nuclear split TurboID by rapamycin induction

Rapamycin (0.1 mg/mL) was added to the culture medium to induce the FKBP-Rapamycin-FRB ternary complex. The formation of this complex allowed configuration of the TurboID N- and C-termini to form active TurboID enzyme.

### 2.5. Assessment of split TurboID biotinylation activity

To assess the biotinylation activity of Split TurboID, the cell-culture experiments summarized in Table were performed. When cells reached 50% confluency, tetracycline was added to the relevant cultures to induce expression of the FKBP-V5-Split TurboID-N-Terminal-NLS protein. After 16 h of expression, rapamycin was added to enable interactions of FKBP and FRB proteins. In addition, 50 μM biotin and 500 mM MgCl_2_ which were required for biotinylation were also added to the cultures. The cultures were incubated for 2 h under standard culture conditions. The cells were then washed with cold PBS and removed from the culture plates by trypsin-EDTA treatment and centrifuged at 1500 × g for 5 min at 4 °C. Cells collected at the bottom of the tubes were frozen and stored in liquid nitrogen.

### 2.6. Immunofluorescence staining

Cells were cultured on poly-L-Lysine coverslips (Corning, USA) to 80% confluency, washed with PBS and fixed with 4% formaldehyde for 20 min before permeabilization with 0.5% Triton-X100 for 2 min. The cells were then incubated with Texas Red conjugated Streptavidin- (Thermo Fisher Sci, USA) for 1 h at room temperature. Nuclei were stained with 4’,6-diamidino-2-phenylindole (DAPI) for 10 min at room temperature. Coverslips were mounted on glass slides using Prolong Diamond Antifade mounting media (Thermo Fisher Sci, USA). The cells were imaged on an Olympus CKx41 microscope equipped with a DP74 digital camera system.

### 2.7. Preparation of protein extracts

The cell pellets were resuspended in RIPA buffer (Thermo Fisher Sci, USA) and homogenized with a bead beater using 0.2 mm stainless steel beads (NextAdvance, Troy, NY). After centrifugation at 10,000 × g at 4 °C for 20 min, the clear supernatants were taken into clean microcentrifuge tubes. Protein concentrations were measured using modified Bradford assay. The protein extracts were snap-frozen in liquid nitrogen and stored at −80 °C.

### 2.8. Enrichment of biotinylated proteins with streptavidin coated beads

Streptavidin-coated magnetic beads (100 μL) (Thermo Scientific, USA) were washed twice with RIPA buffer and 5 mg protein was mixed with magnetic beads. The samples were incubated overnight with rotation at 4 °C and then incubated for 1 h with rotation at room temperature. The beads were then washed twice with 1 mL RIPA lysis buffer, once with 1 mL 1 M KCl, once with 1 mL 0.1 M Na_2_CO_3_, once with 1 mL 2 M urea in 10 mM Tris-HCl (pH 8.0), and twice with 1 mL RIPA lysis buffer. Each wash lasted 30 min with rotation. The beads were then resuspended in 100 μL elution buffer composed of 30 mM biotin, 300 mM NaCl, 2% SDS, 25 mM Tris, pH 7.4 and heated at 95 °C for 5 min. The samples were quickly placed on a magnetic stand and the supernatant without magnetic beads were transferred to a new 1.5 mL microcentrifuge tube.

### 2.9. Verification of enriched biotinylated proteins

Two SDS-PAGE gels were run using 20 μL of protein fractions eluted from streptavidin-coated magnetic beads. One of the SDS-PAGE gels was stained with silver to visualize the proteins while the second one was transferred to a PVDF membrane for western blotting. HRP-conjugated streptavidin was used for signal generation. The bands were visualized on chemo-sensitive X-Ray films.

### 2.10. Tryptic digestion of biotinylated proteins

FASP Protein Digestion Kit (Abcam, USA) was used as recommended by the manufacturer. Briefly, 50 μL eluted proteins were mixed with 300 μL of 8 M urea and the mixture was loaded onto a FASP spin column and centrifuged at 15,000 × g for 15 min. The flow through was removed and the proteins were washed twice with 200 μL of 8 M urea. Then 100 μL of iodoacetamide (9.8 μg/mL in 8 M urea) was added to the spin column and vortexed briefly. The sample was incubated in the dark for 20 min and then centrifuged at 15,000 × g for 15 min, washed twice with 200 μL of 8 M urea and twice with 200 μL of 50 mM ammonium bicarbonate. The FASP spin column was placed into new clean collection tube and 0.5 μg trypsin in 40 μL of 50 mM ammonium bicarbonate solution was added to the surface of the spin column. The spin columns were mildly vortexed and incubated overnight at 37 °C. After completion of the sample digestion, 40 μL of 50 mM ammonium bicarbonate solution was added to the spin column and centrifuged at 15,000 × g for 15 min. A further wash with ammonium bicarbonate was achieved and the peptides were eluted with 50 μ of 500 mM NaCl and concentrated using a SpeedVac. The peptides were resuspended in 0.1% formic acid and their concentrations were measured using Qubit assay (Invitrogen, Q33211).

### 2.11. Protein identification and label-free quantification by nHPLC LC-MS/MS

The peptides were analyzed with nHPLC-MS/MS instrument using an Ultimate 3000 RSLC nano system (Dionex, Thermo Scientific, USA) coupled to a Q-Exactive mass spectrometer (Thermo Scientific, USA). The entire system was controlled by Xcalibur 4.0 software (Thermo Fisher Scientific, USA). High performance liquid chromatography (HPLC) separation was performed using mobile phases of A (0.1% formic acid) and B (80% acetonitrile + 0.1% formic acid). Digested peptides were pre-concentrated and desalted on a trap column. The peptides were then transferred to an Acclaim PepMap RSLC C18 analytical column (75 μm × 25 cm × 2 μm, 100 Å diameter, Thermo Scientific, USA). The gradient applied for separation was 6% for 10 min, 6%–10% B for 60 min, 10%–30% B for 140 min, 30%–50% B for 40 min, 50%–90% for 20 min, 90% B for 10 min and 90%–6% B for 10 min, 6% for 5 min with the flow rate of 300 nL/min in a 305-min total run time. Full scan MS1 spectra were acquired with the following parameters: resolution 70,000, scan range 400–2000 m/z, target automatic gain control (AGC) 3 × E6, maximum injection time 60 ms, spray voltage 2.4 kV. MS/MS analysis was performed by data dependent acquisition selecting the top ten precursor ions. The MS2 analysis composed of collision-induced dissociation (higher-energy collisional dissociation (HCD)) with the following parameters; resolution 17,500, AGC 1E6; maximum injection time 100 ms, isolation window 2.0 m/z normalized and collision energy (NCE) 27. The instrument was calibrated using a standard positive calibrant (LTQ Velos ESI Positive Ion Calibration Solution 88323, Pierce, USA) prior to analysis.

### 2.12. Analysis of mass spectrometry data

The data collected were analyzed with Proteom Discoverer 2.2 software (Thermo Scientific, USA). The following parameters were used for protein identification; Peptide mass tolerance 10 ppm, MS/MS mass tolerance 0.2 Da, mass accuracy 2 ppm, tolerant miscarriage 1, minimum peptide length 6 amino acids, fixed changes cysteine carbamidomethylation, unstable changes methionine oxidation and asparagine deamination. The minimum number of peptides identified for each protein was 1 and all extracted spectra were searched against the UniProt database containing human reference proteome sequence.

### 2.13. Western blotting

Proteins were separated by 12% SDS-PAGE, and electrophoretic transfer of proteins onto PVDF membranes was performed using a semi-dry electrophoretic transfer cell (Bio-Rad, USA) for 30 min at 25 V using 48 mM Tris.Cl buffer at pH 9.2 containing 39 mM glycine, 20% (v/v) methanol and 0.0375 g/L SDS. The membranes were blocked with 5% nonfat dry milk prepared in TBS-T for 1 h at room temperature. The membranes were then incubated overnight at 4 °C with anti-V5 or anti-HA (Thermo Fisher Scentific, USA). HRP-conjugated Streptavidin (Thermo Fisher Scientific, USA) was used to detect biotinylated proteins. A chemiluminescence detection system (Bio-Rad, USA) and ultrasensitive X-ray films (GE Healthcare, USA) were used for band detection.

### 2.14. STRING analysis

STRING analysis was carried out using the Uniprot accession numbers of the identified proteins 1. The search engine option was set to “multiple proteins by names/identifiers” and the organism was specified as Homo sapiens. The retrieved proteins were manually checked to assure that they are all correctly retrieved from the database. Whole genome analysis was the preferred choice. The setting tab was used to change the stringency of the analysis.

## 3. Results

### 3.1. Creation of stable cell line expressing Split TurboID

**Figure 1:**
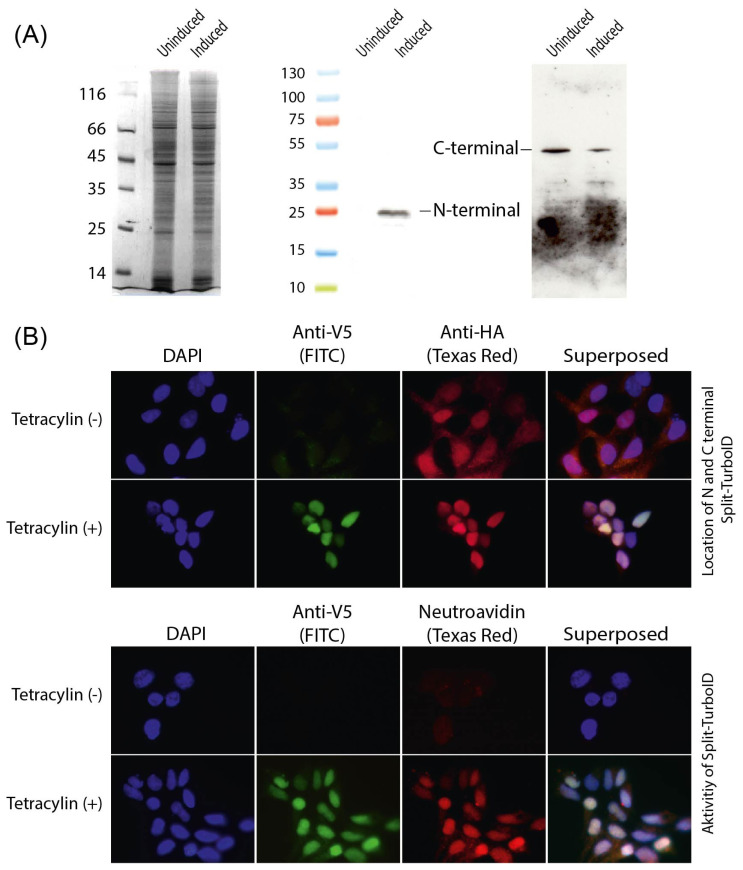
Evaluation of N- and C-termini Split TurboID expressions by the HEK293T-TetR+ monoclonal cell line. (A) Images of a silver-stained SDS-PAGE and Western blots of cell-free extracts. Antibodies raised against HA and V5 tags were used. (B) Immunofluorescence analysis of N- and C-terminal domains of Split TurboID. Antibodies against the V5-tag and HA-tag were used. An FITC-conjugated and a Texas red-conjugated anti-mouse secondar antibodies were used for imaging. (C) Demonstration of biotinylation activity of Split-TurboID by immunofluorescence microscopy. A Texas red conjugated neutravidin was used to visualize nuclear localization of biotinylated proteins. An antibody against the V5-tag and an FITC-conjugated antimouse secondary antibody were used for monitoring localization of active Split-TurboID.

HEK293T-TetR+ cells were sequentially transfected with recombinant vectors to generate a monoclonal cell line expressing the N- and C-termini of Split TurboID. Western blot analysis of protein extracts before and after tetracycline induction using antibodies against HA and V5 tags showed that cells expressed the N-terminus of Split TurboID only after tetracycline induction, while the C-terminus was expressed constitutively (Figure 1A). As predicted, a 26.5 kDa protein band representing the N-terminal domain of Split-TurboID and a 44 kDa band representing the C-terminal domain of Split-TurboID were detected on the blots. Furthermore, immunofluorescence analysis confirmed these findings (Figure 1B). Cells stained with the anti-V5 antibody before tetracycline induction displayed a very weak background stain, whereas staining after tetracycline induction showed clear nuclear localization of the N-terminus of Split TurboID. The same cells showed nuclear staining with an anti-HA tag antibody regardless of tetracycline induction, indicating constitutive expression of the C-terminus of Split TurboID (Figure 1B).

**Figure 2:**
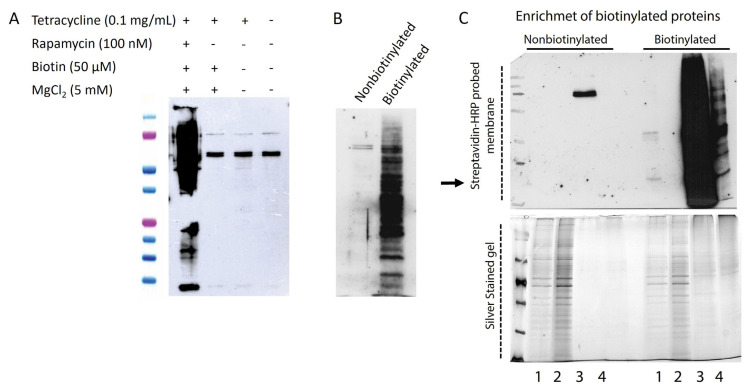
(A) Western blot analysis to assess biotinylation activity of Split TurboID in tetracycline-induced and rapamycin-added cultures. Cell-free extracts were prepared from HEK-293 TetR+ cells to serve as the negative control. (B) Western blot analysis to demonstrate the efficiency of protein enrichment using streptavidin conjugated magnetic beads. (C) Western blot and SDS-PAGE analyses of the enrichment process. The membrane was probed with HRP-conjugated streptavidin to visualize biotinylated proteins. A replica SDS-PAGE gel was run and stained with silver to visualize proteins. Lane numbers: 1, Wash fraction 1; 2, Wash fraction 2; 3, Elution fraction 1; 4, magnetic beads used in the enrichment process. Nonbiotinylated samples that were also subjected to the enrichment process were from a whole cell-lysate that were prepared from nonbiotinylated cells.

To facilitate the formation of an active biotin-ligase enzyme, FKBP and FRB proteins were fused to the N- and C-terminal domains of Split TurboID, respectively. After tetracycline induction and the addition of rapamycin to the culture media, an FKBP-rapamycin-FRB complex formed in the nucleus, triggering the rapamycin-dependent reconstitution of Split TurboID. Biotin was then added to the medium to allow the biotinylation reaction to occur. Immunofluorescence analysis with the anti-V5 antibody showed that biotinylation took place only after the synthesis of the N-terminal domain (Figure 1C). To visualize biotinylated proteins, Texas-red conjugated neutravidin was used. A strong nuclear staining pattern, accompanied by weak cytoplasmic staining, suggested that both resident nuclear proteins and transiently interacting proteins were biotinylated (Figure 1C). Cells were then harvested, and protein extracts were prepared for western blot analysis to assess biotinylation. Biotinylation activity of Split TurboID was detected only in tetracycline-induced and rapamycin-supplemented cultures (Figure 2A).

### 3.2. Enrichment and identification of nuclear proteins

Protein extracts from biotinylated cells were used for nuclear protein enrichment. The enriched proteins underwent western blot analysis with HRP-conjugated streptavidin (Figure 2B). Proteins from culture condition II served as the negative control. An intense signal was observed from a cluster of protein bands in the enriched extracts from biotinylated cells. In contrast, extracts from nonbiotinylated cells lacked this intense signal, indicating the specificity and efficiency of the enrichment method. However, the elution of biotinylated proteins from streptavidin beads was not 100% efficient, as signals from biotinylated proteins were also detected in the lanes where streptavidin-conjugated beads were loaded. To visualize the proteins on the blots, an SDS-PAGE gel was run in parallel and silver stained. Protein bands from both biotinylated and nonbiotinylated samples appeared on the gel. Surprisingly, protein bands were also observed in elution fractions of nonbiotinylated samples, suggesting nonspecific binding of proteins to streptavidin-conjugated beads. Apparently, naturally biotinylated proteins involved in carboxylation reactions might be responsible for some of these protein bands.

**Figure 3:**
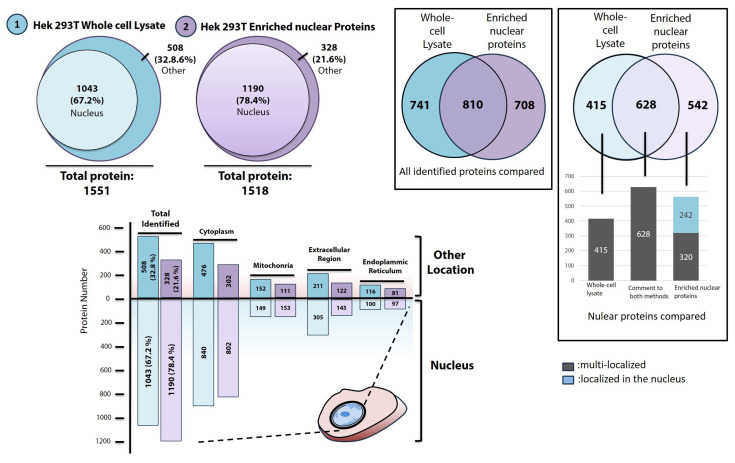
Analysis of the mass-spectrometry data for annotation of nuclear proteins and the proteins colocalize to the nucleus. A protein extract from a whole-cell lysate was analyzed along with a protein extract that was subjected to nuclear protein enrichment. The localizations of each protein were determined using Uniprot and GO annotation databases. Venny 2.1 was used to cross-check the data. The bar graph located in the lower-left corner shows the proteins localized to other compartments. The graph first presents the total number of proteins identified using both nonenriched approaches and enriched. Subsequently, it breaks down the proteins colocalizing to the cytoplasm, mitochondria, extracellular region, and ER. In some instances, a single protein was found in multiple locations (e.g., mitochondria, nucleus, and cytoplasm), and such proteins are included in all relevant bars. To clarify, an example is helpful: the lilac-colored bars represent proteins identified from enriched samples. Light lilac bars indicate proteins colocalizing to the nucleus and mitochondria. Dark lilac-colored bars represent proteins that were not previously reported to localize to the nucleus but identified in this study and known to localize to mitochondria or other compartments. This pattern is consistent across the other bar categories.

Quantitatively, a total of 1518 proteins were identified in the enriched samples. A database search revealed that 1190 (78.4%) of these proteins were exclusively localized to or colocalized to the nucleus (Figure 3). The remaining 328 (21.6%) proteins showed colocalization with other cellular compartments, including mitochondria, Golgi, endoplasmic reticulum, and the extracellular region (Supplementary File 1). For comparison, a similar analysis of a whole-cell lysate that did not undergo nuclear protein enrichment was performed. 1551 proteins, with 1043 (67.2%) localized to or colocalized with the nucleus were identified (Figure 3). The remaining 508 (32.8%) proteins localized to other compartments (Supplementary File 2). When all identified proteins were cross-checked between enriched and nonenriched samples, 810 proteins were found to be shared (Figure 3). When nuclear proteins identified from enriched and nonenriched samples were cross-checked, 628 were shared by both samples, 415 were present only in the nonenriched sample, and 542 were present only in the enriched sample. Further analysis showed that nuclear proteins from nonenriched samples colocalized with other organelles or subcellular structures, implying the presence of organelle contamination. On the other hand, 242 (45%) of nuclear proteins from enriched samples were localized exclusively to the nucleus, indicating that nuclear proteins were more reliably enriched (Supplementary File 3).

**Figure 4:**
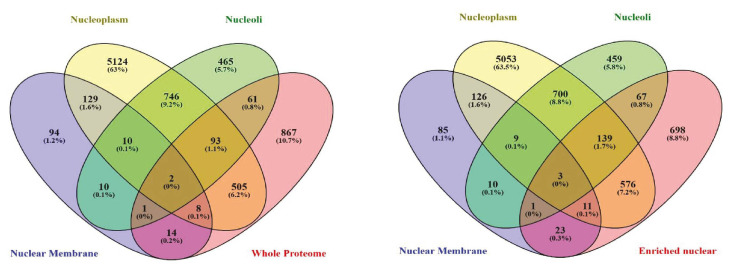
Venny Diagrams showing common nuclear proteins listed in Human Protein Atlas and identified in this study. Venny 2.1 was used to cross-check the data.

The Human Protein Atlas (HPA) database categorizes nuclear proteins into nuclear membrane, nucleoli, and nucleoplasm proteins. We retrieved these proteins and cross-checked our lists. Of the proteins identified, 820 of the proteins present in the enriched nuclear protein sample were also reported in HPA database as nuclear proteins (Figure 4A). Surprisingly, however, 698 proteins were not present in HPA database. Searching these 698 proteins in Uniprot and GO databases showed that many of these proteins were annotated as nuclear proteins. A similar analysis on the whole-cell lysate yielded similar results, indicating that the HPA database alone is not sufficient for annotating subcellular localization of proteins (Figure 4B).

## 4. Discussion

Determining protein localization is crucial for understanding the physiological roles of proteins. However, this task poses a significant challenge in cell biology, as it is difficult to determine the localization of numerous proteins on a large scale (Emanuelsson et al., 2000; Simpson et al., 2000; Hua and Sun, 2001; Dunkley et al., 2004; Elzek et al., 2021). Traditional subcellular fractionation (SF) techniques have been employed to enrich subcellular compartments and identify proteins using mass spectrometry for annotation of spatial distribution (Fialka et al., 1997; Cox and Emili, 2006; Lee et al., 2010; Masuda et al., 2020). Although SF is a powerful technique, isolating target proteins without contamination from other cellular components remains a considerable challenge. To address these limitations, various high-throughput techniques have been developed and utilized. These include genome-wide ORF-GFP tagging, gene-trap screens, high throughput localization assays using myc-tags, and mass spectrometry-based proteomics (Holzschu et al., 1997; Davis, 2004; Meurer et al., 2018). These methods have made it possible to determine localizations of many proteins, but due to tendency of proteins to display multiple localization, they are not able to fully capture the dynamic nature of cells (Mueller et al., 2004; Simha et al., 2015; Ota et al., 2016). Continued efforts and the development of innovative techniques are essential to overcome these limitations and gain deeper insights into the spatial distribution and functions of proteins.

In this study, we focused on nuclear proteins, as the nucleus plays a crucial role in maintaining genetic integrity, regulating gene expression, orchestrating cellular activities, and ensuring proper cell division and differentiation (Zidovska, 2020). These functions are pivotal to the overall health and functioning of eukaryotic cells, rendering nucleus an indispensable organelle for the survival and proper operation of organisms. Moreover, the cell nucleus is intricately linked to various human diseases, such as cancer, genetic disorders, neurodegenerative diseases and others (Zwerger et al., 2011). Therefore, comprehending the role of nucleus in disease processes is imperative for the development of targeted therapies and effective treatments to address these conditions.

The traditional method for isolating nuclei involves the use of nonionic detergents (Anand and Chaudhuri, 2023), which can lead to the aggregation of nuclei and disruption of nuclear membranes, resulting in a loss of nuclear protein content. To overcome these limitations, detergent-free methods have been described in the literature (Eski et al., 2020). However, these methods have also faced challenges due to impurities from contaminating organelles and loss of nuclear material during their application. In recent years, proximity labeling (PL) has emerged as an alternative approach to map proteins to their subcellular locations within living cells. For this purpose, researchers expressed TurboID in living cells, allowing it to label proteins within the organelles (Cho et al., 2020a). After biotinylation, nuclear proteins were isolated using streptavidin-conjugated beads, and the biotinylated proteins were analyzed with mass spectrometry. This approach is superior over many nuclear protein enrichment methods but still suffers from contamination by proteins from other subcellular compartments. One issue is that after transfection, TurboID synthesis begins in the cytoplasm, and the active enzyme is then transferred to the organelle of interest, potentially biotinylating cytoplasmic proteins along the way, which leads to confusion regarding their spatial distribution. To address this concern and develop a tightly-controlled approach, we have used the enzyme Split TurboID to improve the specificity of labeling proteins within nucleus (Cho et al., 2020b). Split TurboID is a low affinity enzyme and catalyze biotinylation in a biotin-dependent manner.

In here, we modified the Split TurboID-based biotinylation approach to achieve a tighter control. By controlling the expression of the N-terminal domain of Split-TurboID, we ensured that the active enzyme was formed exclusively within the nucleus. Further regulation of the formation of active Split-TurboID was achieved by rapamycin. The overall system was monitored by immunofluorescence staining of the cells using V5 and HA epitope tags. Additionally, western blot analysis was performed with antibodies specific to these tags, confirming the presence of split-TurboID within the nucleus. Our approach effectively minimized background noise arising from nonspecific labeling or imperfect localization of Split-TurboID.

By using the approach described here, we were able to identify proteins predominantly localized to the nucleus (15% of the identified proteins). STRING analysis performed with these proteins predicted cluster of networks that are exclusively involved in nucleic acid metabolic processes (FDR: 1.12e–99). The biological pathways associated with these proteins were also revealed and they were associated with splicosome (FDR:7.06e–31), ribosome biogenesis (FDR: 2.09e–15), mismatch repair (FDR: 1.01ee–15) and DNA replication (FDR: 1.01e-15). When proteins colocalized with other cellular organelles were subjected to STRING analysis, cluster of networks associated with many metabolic processes including gene expression, protein translation and protein transport to nucleus were identified.

### 4.1. Mitochondrial proteins colocalized to the nucleus

Mitochondrial functions are tightly regulated by nuclear activity, which requires extensive communication between these organelles. In fact, the translocation of mitochondrial proteins into nuclei represents the simplest and most direct means of retrograde communication between the two organelles (Monaghan and Whitmarsh, 2015; Gonzalez-Arzola et al., 2019). Literature reports explain how retrograde communication occurs between mitochondria and the nucleus (Soledad et al., 2019). In some cases, a protein may contain both a mitochondrial targeting sequence (MTS) and a nuclear localization signal. However, for most proteins, this may not be the case. Mitochondrial exosomes or other types of vesicles may be used to transport proteins to the nucleus. Alternatively, leakage or breakdown of the mitochondrial membrane could allow proteins to enter the nucleus. In most instances, proteins relocate to the nucleus only when there is a need to protect nuclear integrity within cells. Depending on the cellular requirements, the distribution of these proteins between compartments may be rapidly modulated. For instance, Parkin, an E3 ubiquitin ligase, typically functions in the cytoplasm to mark proteins for degradation in proteasomes (Durcan and Fon, 2015). However, when mitochondria are damaged, Parkin is recruited to the damaged mitochondria and functions to mark the compromised organelle. Interestingly, this same protein is also found in the nucleus, displaying an eclipsed distribution and functions as a tumor suppressor protein (Ayimugu et al., 2020; Rouland et al., 2021).

Mitochondria form contact sites with nucleus to drive cellular adaptation to stress by retro-communication (Desai et al., 2020). The physical nature of this contact suggests the absence of membrane fusion, with the membranes actively held together by tether molecules. One such tethering protein was discovered to be Cnm1, for Contact Nucleus Mitochondria 1 (Eisenberg-Bord et al., 2021). In line with being a tether component, overexpression of Cnm1 resulted in mitochondrial crowding around the nucleus, indicating that this protein can mediate active recruitment of mitochondria to be in contact with the nucleus. Recent findings have highlighted the necessity for mitochondria to overexpress a protein, TSPO, to facilitate this contact (Desai et al., 2020). In addition to TSPO, the involvement of other proteins such as the A-kinase anchoring protein acyl–coenzyme A binding domain containing 3 (ACBD3), protein kinase A (PKA), and A-kinase-anchoring protein (AKAP95) has been identified as crucial in tethering mitochondria to the nucleus (Ovciarikova et al., 2022). The proximity between these organelles is postulated to enhance cell survival through a mitochondrial retrograde response. The proteins identified in this study have not been extensively evaluated in the literature regarding their participation in establishing contact between mitochondria and the nucleus. Nevertheless, they should inspire the field to explore whether the cellular processes depend on the exchange of these proteins between the nucleus and the mitochondrion.

### 4.2. The endoplasmic reticulum proteins colocalized to the nucleus

ER is a network of membrane-enclosed tubules and sacs (cisternae) that extend from the nuclear membrane throughout the cytoplasm. We identified proteins that dually localized to the nucleus and the Endoplasmic reticulum. The human protein atlas reported localization of one of these proteins to the nucleus based on immunofluorescence microscopy analysis. STRING analysis with these dually localized proteins underlined ER-associated degradation (ERAD) pathway. Some components of the ERAD machinery, including certain ubiquitin ligases, might be known to translocate to the nucleus under specific conditions. For example, HRD1, an ER membrane-embedded ubiquitin ligase involved in ERAD, has been shown to translocate to the nucleus in response to ER stress (Doroudgar et al., 2015; Fenech et al., 2020). The nuclear translocation of ERAD components might have regulatory roles in coordinating the cellular response to ER stress. ERAD is a process primarily localized to the endoplasmic reticulum, there are connections between ERAD and the nucleus. These connections involve transcriptional regulation of ERAD components, nuclear translocation of certain ERAD-related proteins, and nucleocytoplasmic shuttling of factors that participate in both ER stress responses and ERAD. These mechanisms contribute to the integrated cellular response to ER stress, ensuring the maintenance of protein homeostasis within the cell.

### 4.3. Plasma membrane and extracellular matrix proteins colocalized to the nucleus

Proteins that were not previously known to localize in the cell membrane were identified in this study and considered to be colocalized between the cell nucleus and the plasma membrane. STRING analysis of these proteins did not reveal any functional protein association network. Based on the HPA, 283 proteins are supposed to colocalize between the nucleoplasm and plasma membrane, 7 proteins colocalize between the nuclear membrane and plasma membrane, and 20 proteins colocalize between nucleoli and the plasma membrane. Some of the proteins identified in this study were not previously reported to colocalize with the nucleus. Knowing their localization is important to attribute them to biological functions in health and disease.

### 4.4. Cytosolic proteins colocalized to the nucleus

STRING analysis of the proteins that are listed as cytosolic in all three data banks but were identified in our nuclear fractions underlined cytoplasmic translation events. It has long been known that ribosome biogenesis requires nuclear translocation of ribosomal proteins from their site of synthesis in the cytoplasm to the nucleus. The mechanism of transport for most ribosomal proteins are known. Multiple transport receptors, i.g., karyopherins or importins participate transport of ribosomal proteins (Plafker and Macara, 2002). In this study, several ribosomal proteins in the nuclear fractions were identified.

The subcellular location of T-complex protein-1 subunit gamma (TRiC) was detected in the nuclear fraction although this protein was not listed as a nuclear or nuclear-associated protein in all three protein data banks that were used in this study. However, our observation agrees with the physiological function of TRiC, which is required for folding the telomerase cofactor TCAB1, that controls trafficking of telomerase and small Cajal body RNAs (scaRNAs) (Freund et al., 2014). The other protein that was detected in nuclear fraction was immunoglobulin-binding protein 1 This protein is involved in signal transduction, and regulation of the catalytic activity of the phosphatases PP2A, PP4, and PP6 by protecting their partially folded catalytic subunits from degradative polyubiquitination (Jiang et al., 2013). The proteins PP2A, PP4, and PP6 are nuclear-associated proteins implying that their regulatory protein, immunoglobulin-binding protein 1, might also associate with the nucleus as we demonstrated in this study. F-box only protein 40 (FBX4) was detected as a nuclear or nuclear-associated protein in our study, although it is listed as a cytoplasmic protein in two of the three data banks. FBX4 mediates ubiquitination and subsequent proteasomal degradation of target proteins e.g., CCND1 and TERF1 (Lee et al., 2006; Barbash et al., 2008). Both CCND1 and TERF1 are localized to nucleus justifying that FBX4 might also be a nuclear-associated protein. The last cytoplasmic protein that we identified in our nuclear fractions was lysophospholipase-like protein-1. This protein was reported to have depalmitoylating activity toward KCNMA1 (Tian et al., 2012). Neither lysophospholipase-like protein-1 nor its substrate is associated with nucleus or nuclear membranes. Both proteins are localized to the cell membrane. The physiological function of this protein in the cell-membrane does not justify our observation of it in nuclear fractions. We suppose that lysophospholipase-like protein-1 might be temporarily associating with nuclear membranes and may interact with channel proteins as it does in the cell membrane.

## 5. Conclusion

In eukaryotic cells, proteins are dynamic entities, continuously moving between different locations. This dynamic behavior often results in their presence in multiple cellular compartments. Interestingly, some proteins exhibit a “moonlighting” effect, appearing in unexpected locations (Rodriguez-Saavedra et al., 2021). A notable example is GAPDH, a glycolytic enzyme typically localized in the cytoplasm, yet also found in the nucleus. In our study, we observed several proteins colocalizing with the nucleus. Many of these proteins appear to be bona fide nuclear residents, as supported by the subcellular location data published in the Human Protein Atlas 2. However, our list may also include de javu protein identifications, despite the stringent washing conditions employed to minimize false positives (Petrak et al., 2008). Nonetheless, the provided list represents candidate proteins requiring individual validation to confirm their nuclear localization.

The presented study focused on addressing the challenges associated with determining the localization of proteins, with a particular emphasis on nuclear proteins. The conventional methods face limitations in isolating target proteins without contamination. To overcome these challenges, we employed a modified Split TurboID-based biotinylation approach, offering enhanced specificity for labeling proteins within the nucleus. Proteins that were not previously associated with nucleus were identified. In addition, proteins with dual localization were also identified prompting further exploration into the intricate communication between organelles. In essence, we believe that this study helped advance the methodology for protein localization studies and also provided novel insights into the diverse roles of proteins in the nucleus, mitochondria, ER, and cell membrane. Continued efforts in developing innovative techniques, combined with comprehensive exploration of protein localization, will undoubtedly contribute to a more detailed understanding of cellular functions.

## Supplementary Information







## Figures and Tables

**Table:** Design of the in vitro cell culture experiments to assess the formation of active Split-TurboID upon tetracycline induction.

Components Added	Condition I (Biotinylation ^+^)	Condition II (Biotinylation ^−^)	Condition III (Biotinylation ^−^)	Condition IV (Biotinylation ^−^)
Tetracycline (0.1mg/ml)	**+**	**+**	**+**	−
Rapamycin (100 nM)	**+**	−	−	−
Biotin (50 μM)	**+**	**+**	−	−
MgCl_2_ (5 mM)	**+**	**+**	−	−

## Data Availability

Raw Mass-spec data will be provided if asked from the corresponding author.

## Footnotes

1 STRING (2024). Multiple Proteins Search [online]. Website https://string-db.org/cgi/input?sessionId=bXSy8ZE2J6HM&input_page_active_form=multiple_sequences [accessed 3 March 2024].

2 Human Protein Atlas (2024). The Subcellular Resource [online]. Website https://www.proteinatlas.org/humanproteome/subcellular [accessed 5 March 2024].
